# Syndrome de cimeterre: à propos d’un cas et revue de la literature

**DOI:** 10.11604/pamj.2016.25.37.10545

**Published:** 2016-09-28

**Authors:** Ibtihale Benjouad, Ikram Taam, Khaoula El Ataouna, Mohamed Mahi, Touriya Amil, Rachida Saouab

**Affiliations:** 1 Service d’Imagerie Médicale, Hôpital Militaire d’Instruction Mohammed V, Rabat, Maroc; 2 Service de Pneumologie, Hôpital Militaire d’Instruction Mohammed V, Rabat, Maroc

**Keywords:** Syndrome de cimeterre, anomalies cardio-pulmonaires, angioscanner tomodensitométrie, Scimitar syndrome, cardiopulmonary anomalies, CT

## Abstract

Le syndrome du cimeterre ou syndrome veino-lobaire de Felson est une pathologie très rare, caractérisée par l’association d'anomalies cardio-pulmonaires notamment un retour veineux pulmonaire droit anormal, situé le plus souvent dans la veine cave inférieure. Nous présentons une observation originale d’unnourrisson de sexe féminin, âgée de six mois, qui s’est présenté pour une dyspnée aigue. Le diagnostic a été suspecté sur la radiographie thoracique et confirmé à la tomodensitométrie qui mettait en evidence une grosse veine pulmonaire droite unique se jetant au niveau de l’oreillette droite associée à une dextrocardie et une séquestration pulmonaire. Le pronostic est lié à l’importance du shunt gauche-droit et aux malformations associées.

## Abstract (en)

Scimitar syndrome or veino-lobaire syndrome (term coined by Felson) is a very rare disease characterized by the combination of cardiopulmonary anomalies, in particular an anomalous right pulmonary venous return, located mostly in the inferior vena cava. We here report the original case of a 6-month-old female infant presenting with acute dyspnea. The diagnosis was suspected on the basis of thoracic radiograph and was confirmed by tomodensitometry which showed a large single right pulmonary vein draining into the right atrium associated with dextrocardia and pulmonary sequestration. The prognosis was based on the size of the left-right shunt and related malformations.

## Introduction

Le syndrome de cimeterre ou syndrome de Halasz est une maladie exceptionnelle caractérisée par un retour veineux pulmonaire droit anormal partiel ou total. La veine pulmonaire supérieure droite ou les deux veines droites se drainent alors dans la veine cave supérieure, dans la veine azygos, dans la veine cave inférieure ou plus rarement directement dans l’oreillette droite.

## Patient et observation

**Figure 1:**
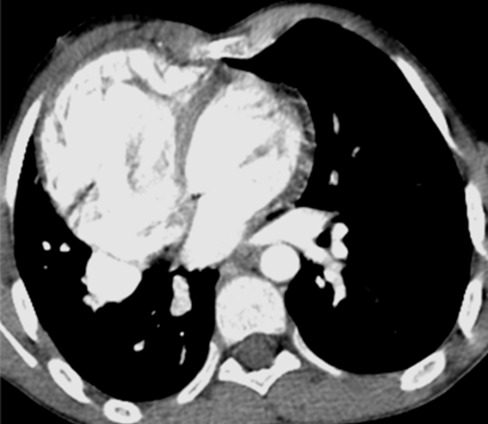
Coupe tomodensitométrique axiale, après injection du produit de contraste montrant une veine pulmonaire droite unique qui se jette dans l’oreillette droite

**Figure 2:**
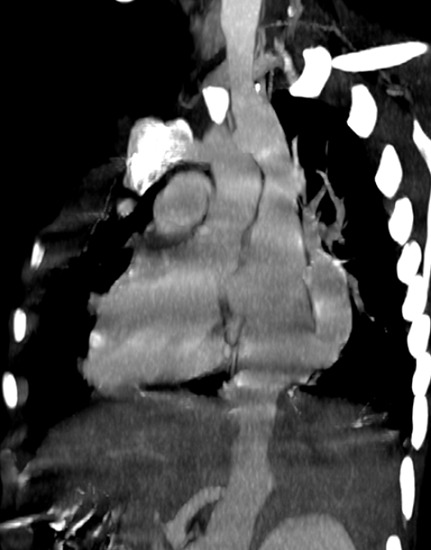
Coupe tomodensitométrique en reconstruction sagittale après injection du produit de contraste, objectivant une veine pulmonaire droite unique qui se jette dans l’oreillette droite. On note l’aspect en sabre ou en “cimeterre” de la veine pulmonaire droite

**Figure 3:**
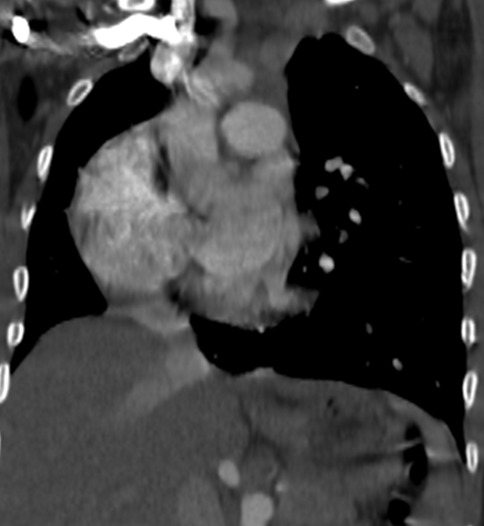
Reconstruction tomodensitométrique coronale montrant la dextrocardie

**Figure 4:**
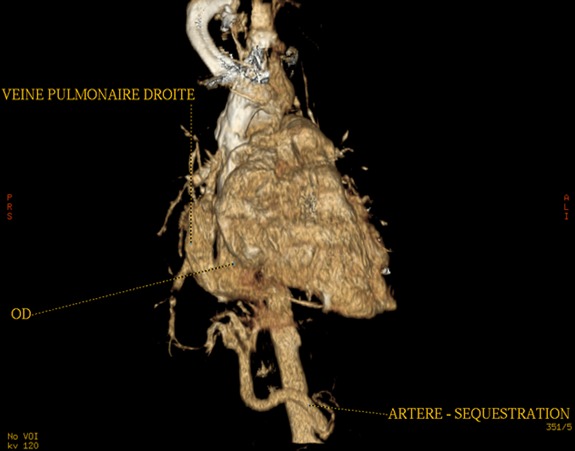
Reconstruction tomodensitométrique volumique montrant les malformations associées. On note une artère systémique naissant du tronc cœliaque et se dirigeant vers la séquestration pulmonaire basale droite

Il s’agit d’un enfant âgé de six mois, de sexe féminin, qui a été hospitalisée pour dyspnée aiguë sifflante. Le traitement symptomatique d’une bronchiolite a été instauré, mais l’évolution a été marquée par la persistance de la détresse respiratoire et des difficultés alimentaires. La radiographie du thorax a été réalisée et a objectivé une cardiomégalie ainsi qu’une opacité pulmonaire postéro-basale droite. Le complément d’angioscanner thoracique (Figure 1, Figure 2, Figure 3) a montré une grosse veine pulmonaire droite unique se jetant au niveau de l’oreillette droite, associée à une dextrocardie et cardiomégalie. On a aussi noté une branche artérielle prenant naissance au niveau du tronc cœliaque et allant vers la base pulmonaire droite en rapport avec une sequestration pulmonaire intra lobaire S1 (Figure 4).

## Discussion

Le syndrome du cimeterre est un groupe complexe d’anomalies veino-pulmonaires regroupées également sous les termes de syndrome veino-lobaire de Felson ou syndrome de Halasz connu depuis plus d’un siècle [1]. Ce syndrome correspond avant tout à un retour veineux pulmonaire droit anormal (RVPA) qui s’associe classiquement à d’autres malformations telles : une dextro-position cardiaque par rotation, un petit poumon droit volontiers bi lobaire, une séquestration broncho-pulmonaire et/ou des malformations cardiaques, notamment une communication inter-auriculaire (CIA) [2]. Le nom de ce syndrome revient à l’aspect en sabre turc ou « cimeterre » du RVPA droit sur la radiographie thoracique de face. Ce RVPA draine l’ensemble ou une partie du retour veineux du poumon droit dans la veine cave inférieure supra hépatique, dans l’atrium droit juste au-dessus de l’abouchement de la veine cave inférieure, parfois dans une veine sus-hépatique. Sa prévalence est estimée entre 1/100 000 et 1/33 000 naissances vivantes. La maladie semble toucher majoritairement les filles. L´étiologie n´est pas complètement comprise. Chez plusieurs patients avec un retour pulmonaire veineux totalement anormal, le locus du gène a été localisé sur le chromosome 4q12. L’association d’un RVPA type cimeterre à une séquestration est décrite chez 50 % des patients ayant ce syndrome [3]. Le plus souvent, la séquestration pulmonaire est de type I de Pryce. L’artère systémique aberrante naît le plus souvent de la partie inférieure de l’aorte thoracique descendante ou de la partie initiale de l’aorte abdominale [4]. La recherche d’une séquestration est systématique avant d’entreprendre un éventuel traitement chirurgical du RVPA. La méconnaissance de la vascularisation artérielle aberrante de la zone séquestrée pouvant entraîner des risques hémorragiques majeurs [5]. L’expression clinique est très variable, allant d’une intolérance dès les premiers jours de vie à une découverte fortuite chez l’adulte ; elle dépend de l’importance de l’hypoplasie pulmonaire et de la fistule artério-veineuse à travers la séquestration [6]. Dans la majorité des cas, il se manifeste durant la période néonatale par une insuffisance cardiaque congestive due habituellement à une hypertension pulmonaire et une insuffisance respiratoire. Les autres complications cliniques majeures sont les infections pulmonaires favorisées par la distorsion architecturale du poumon hypoplasique ; l’hémoptysie et l’hémothorax sont favorisés par la vascularisation systémique du poumon séquestré [4]. Le diagnostic est évoqué sur la radiographie du thorax de face devant la présence d’une opacité arciforme basale droite, allant de la région hilaire jusqu’à la coupole diaphragmatique. Le bilan complet d’un syndrome du cimeterre comporte d’une part un bilan morphologique à la recherche des anomalies associées et d’autre part un bilan fonctionnel pour évaluer la surcharge du cœur droit par le RVPA. L’examen de référence pour l’analyse morphologique du parenchyme pulmonaire, de l’arbre bronchique, de l’anatomie vasculaire pulmonaire et systémique est aujourd’hui l’angio-TDM volumique. Le bilan fonctionnel, avec cotation de l’importance du shunt, en mesurant le débit dans le tronc de l’artère pulmonaire (Dp) et le débit du cœur gauche (Dg), est réalisé classiquement par échocardiographie. Ces dernières années, l’enregistrement du flux par IRM en contraste de phase, peut avoir une place dans l’évaluation de ces deux débits [7], en comparant le débit artériel pulmonaire et le débit aortique. Le traitement curatif du syndrome du cimeterre implique dans certains cas une chirurgie lourde associant une réimplantation de la veine pulmonaire dans l’atrium gauche et une chirurgie des autres éventuelles malformations cardiovasculaires associées et notamment la chirurgie d’une séquestration pulmonaire [3] et la fermeture d’une CIA. L’indication chirurgicale du RVPA repose sur la constatation d’une insuffisance cardiaque droite et d’un rapport Dp/Dg supérieur à deux. En cas d’hémoptysie compliquant la séquestration, un traitement isolé par vaso-occlusion radiologique de l’artère séquestrée peut être proposé en urgence dans les séquestrations purement vasculaires [8]. En cas de RVPA partiel du lobe inférieur droit avec séquestration compliquée d’hémoptysie et de surinfections, une lobectomie inférieure peut être discutée en l’absence d’anomalie scissurale qui pourrait rendre cette lobectomie difficile; le RVPA est alors retiré et traité.

## Conclusion

Le syndrome du cimeterre est une maladie exceptionnelle, méconnue, dont l’expression clinique est insidieuse et non spécifique. La radiographie thoracique permet souvent d'évoquer le diagnostic qui sera confirmé par l’angioscanner thoracique, l'angiographie pulmonaire ou l'IRM. Aucun traitement n'est nécessaire chez les patients asymptomatiques ; un traitement chirurgical pourra cependant être proposé en cas de shunt gauche droit sévère, de séquestre ou d'infections pulmonaires à répétition.
